# Socioeconomic Status and Its Relation to Hypertension in Rural Nepal

**DOI:** 10.1155/2021/5542438

**Published:** 2021-08-28

**Authors:** Sanju Bhattarai, Birgit Tandstad, Archana Shrestha, Biraj Karmacharya, Abhijit Sen

**Affiliations:** ^1^Department of Community Programs, Dhulikhel Hospital Kathmandu University Hospital, Dhulikhel, Nepal; ^2^Department of Public Health and Nursing, Norwegian University of Science and Technology, Trondheim, Norway; ^3^Department of Public Health, Kathmandu University School of Medical Sciences, Dhulikhel, Nepal; ^4^Institute of Implementation Science and Health, Kathmandu, Nepal; ^5^Department of Chronic Disease Epidemiology Center of Methods for Implementation and Prevention Science, Yale School of Public Health, New Haven, USA; ^6^Oral Health Services and Research Center, (TkMidt), Trondheim, Norway

## Abstract

**Introduction:**

Hypertension and its association with socioeconomic positions are well established. However, the gradient of these relationships and the mediating role of lifestyle factors among rural population in low- and middle-income countries such as Nepal are not fully understood. We sought to assess the association between socioeconomic factors (education, income, and employment status) and hypertension. Also, we assessed whether the effect of education and income level on hypertension was mediated by lifestyle factors.

**Methods:**

This cross-sectional study was conducted among 260 participants aged ≥18 years attending a rural health center in Dolakha, Nepal. Self-reported data on demographic, socioeconomic, and lifestyle factors were collected, and blood pressure, weight, and height were measured for all study participants. Those with systolic blood pressure ≥140 mm Hg or diastolic blood pressure ≥90 mm Hg or administrating high blood pressure-lowering medicines were regarded as hypertensives. Poisson regression models were used to estimate the prevalence ratios and corresponding 95% confidence intervals to assess the association between socioeconomic factors and hypertension. We explored mediation, using the medeff command in Stata for causal mediation analysis of nonlinear models.

**Results:**

Of the 50 hypertensive participants, sixty percent were aware of their status. The age-standardized prevalence of hypertension was two times higher for those with higher education or high-income category. Compared to low-income and unemployed groups, the prevalence ratio of hypertension was 1.33 and 2.26 times more for those belonging to the high-income and employed groups, respectively. No evidence of mediation by lifestyle factors was observed between socioeconomic status and hypertension.

**Conclusions:**

Socioeconomic positions were positively associated with hypertension prevalence in rural Nepal. Further studies using longitudinal settings are necessary to validate our findings especially in low- and middle-income countries such as Nepal.

## 1. Introduction

Hypertension is one of the major risk factors for cardiovascular diseases (CVDs) and all-cause mortality globally [1] disproportionately affecting middle-aged individuals in low- and middle-income countries (LMICs) including Nepal. The economic implication of CVDs is huge; they cost LMICs USD 3.7 trillion between 2011 and 2015, approximately 2% of the gross domestic product across LMICs [2]. A national survey in Nepal reported a prevalence of 24.5% [3], ranging from 12% in rural populations [4, 5] to 29% in semiurban population [6], and this percentage will keep increasing every year. Therefore, in resource-constrained countries such as Nepal, effective management of hypertension is imperative for reducing CVD events and associated economic burdens [7].

Socioeconomic status (SES) is a strong predictor for hypertension, its awareness, and adherence to control measures [8]. In high-income nations, individuals with a lower level of education and income have been associated with an increased risk of hypertension [9–11]. However, in LMIC, this association is complex. For instance, the prevalence of hypertension was higher among low SES groups in Brazil [12, 13] and Peru [14], while greater among high SES groups in South Asian countries [14–18]. Studies from Nepal have also reported an elevated blood pressure among affluent individuals [19], whereas highly educated individuals [4, 5] and men doing labor-intensive work had normal blood pressure. The possible reasons for the low prevalence of hypertension among higher SES groups in LMICs might be due to increasing health awareness [20, 21], lower psychological stressors [20], and better accessibility of and adherence to medical treatment [22] among highly educated groups. On the other hand, the higher prevalence seen among low SES groups might be due to sedentary lifestyle choices [23, 24], which are rising due to urbanization and globalization in LMICs [25] such as Nepal.

The inconsistencies in the SES and hypertension association warrant exploration of potential modifiable mediators. Lifestyle factors such as body mass index (BMI), alcohol intake, physical activity, and smoking are commonly viewed as mediators between SES and health and that healthy lifestyle might attenuate the socioeconomic inequities in health [26, 27]. Studies from high-income countries suggest that variations in BMI, smoking, and alcohol between different SES groups [28] account for substantial proportion of inequalities in hypertension [10, 29]. However, these roles are not adequately explored in low-income settings where health system capacities and disease profiles are different. Most earlier hypertension studies in Nepal have focused on estimation of prevalence among urban and semiurban areas. To the best of our knowledge, so far, no study has documented the mediating role of lifestyle factors in SES and hypertension associations in rural Nepal. The primary objective of this study was to assess the temporal association between SES and prevalence of hypertension among patients visiting Kirnetar Health Center in rural Nepal. Furthermore, we assessed whether the effect of these socioeconomic factors on hypertension was mediated by lifestyle factors.

## 2. Methods

Study setting: the study was conducted at Kirnetar Health Center in the rural village of Dolakha district in Nepal. The health center was established in 2012, and it serves the population from eight nearby villages providing primary level health services six days a week, including 24-hour emergency services.

Study design and population: a cross-sectional study was conducted among individuals who visited the Kirnetar Health Center for clinical examination or to purchase medicine from 27.10.2016 through 01.12.2016. Voluntary participants above 18 years were included, whereas pregnant women were excluded from the study.

Sample size: the sample size of 260 participants was estimated using the Raosoft sample size calculator at 80% power and 5% critical limit (95% confidence interval). The estimated margin of error with this sample size was 200 (6.48%) and 300 (5.10%).

Data collection: all recruited voluntary participants were interviewed by the trained enumerators. The self-reported information on demographic, socioeconomic, clinical history, lifestyle, and dietary factors were collected using a validated STEPS Questionnaire, developed by the World Health Organization (WHO) [30]. The GT-702 Fully Automatic Arm Style Digital Blood Pressure Monitor was used to measure the participants' systolic blood pressure (SBP) and diastolic blood pressure (DBP) twice (15 minutes apart) in a sitting position. The mean of two systolic and diastolic blood pressure measures was considered for the analysis. Participants stood on the electronic scale (Bosch Electronic Scale PPWA4201) placed on the flat floor to measure weight (in kgs), and for height, lineal measurement of top point of the participants' head when standing on their heels and head against the measuring tape placed on the wall was measured to nearest 0.5 cm. Body mass index was calculated as weight in kilograms divided by height in metres squared.

### 2.1. Outcome

Participants with SBP of 140 mm Hg or higher, or DBP of 90 mm Hg or higher, or those taking hypertensive medication prior to the interview were defined as hypertensive.

Hypertension awareness and treatment: participants who were informed by a doctor/health worker about their raised blood pressure were recognized as aware of blood pressure status. Those who reported having ever used antihypertensives were considered on treatment.

### 2.2. Exposures

Income: the per capita annual income was calculated by asking the total combined household income (in Nepali rupees) in the year preceding the survey and dividing it by the total number of household members. Income was categorized into tertiles (low, middle, and high).

Education: participants who reported that they did not attend school were confined to the “no formal education” group, those who had at least one year of formal school including those not completing high school were confined to the “less than high school” group, and those who had completed high school or beyond were confined to “high school and above” group.

Employment status: the variable was classified into three categories: farming (agricultural task), employed (government/nongovernment employees and self-employed persons), and unemployed (retired, students, unpaid, unable to work, unemployed, and homemakers).

### 2.3. Covariates

Sociodemographic variables include age (in years), gender (males and females), marital status (yes and no), and ethnicity (Dalit, Brahmin, Chettri, and others). Lifestyle-related variables include both smoke or smokeless tobacco use (categorized as never-users, current, and former users); alcohol intake (drinking <1 glass/week, 1–3 glasses/week, and >3 standard drinks/week were categorized as “low drinkers,” “moderate drinkers,” and “heavy drinkers,” respectively); physical activity was assessed using the Global Physical Activity Questionnaire [31] (≥600 metabolic equivalent minutes (MET) and <600 MET was categorized as adequate and inadequate, respectively); fruits and vegetables servings (<2, 2–4, and >4 servings per day); and body mass index (<18.5 kg/m^2^, 18.5–24.9 kg/m^2^, 25.0–25.9 kg/m^2^, and ≥30.0 kg/m^2^ categorized as underweight, normal, overweight, and obese, respectively) [32].

### 2.4. Statistical Analysis

The descriptives were presented as frequencies and percentages for categorical variables and mean and standard deviation (SD) for continuous variables. Age-standardized hypertension prevalence was calculated using the WHO standard population. Prevalence ratio (PR) and corresponding 95% confidence intervals were computed to assess the association between socioeconomic positions and the prevalence of hypertensive using Poisson regression models with robust standarderrors [33]. We fitted Poisson regression to estimate PR because odds ratio does not give a good approximation of the risk in cross-section data with high prevalence of outcome [34]. Two models were constructed. Model 1 was unadjusted, and Model 2 was adjusted for confounders such as age, gender, marital status, and ethnicity.

**Figure 1:**
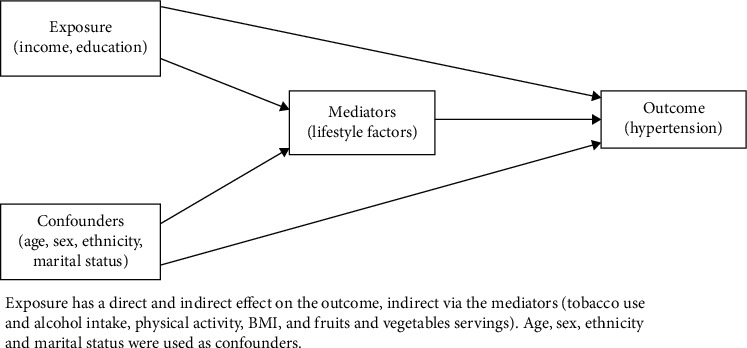
Hypothesized causal diagram. Exposure has a direct and indirect effect on the outcome, indirect via the mediators (tobacco use and alcohol intake, physical activity, BMI, and fruits and vegetables servings). Age, sex, ethnicity, and marital status are used as confounders.

Based on evidence [35–39], we hypothesized that the causal effect of SES (education and income) on hypertension is mediated via lifestyle-related factors such as tobacco, alcohol intake, physical activity, BMI, and fruits and vegetable intake as illustrated in Figure 1. We explored mediation, using the medeff command in Stata for causal mediation analysis of nonlinear models [40, 41]. For each mediator, two regression models were fitted. First, the mediator was regressed on the exposure (income and education), and second, the outcome (hypertension) was regressed on the exposure and mediator variable (one by one). Predictions from these models were then used within a Monte-Carlo framework to calculate estimates for total, indirect, and direct effects [42]. This process decomposes the total effect of SES variables on hypertension (i.e., the probability of being hypertensive per unit change in income and education) into an indirect effect (i.e., mediated effect statistically explained by variation in the mediator path connecting SES and hypertension) and a direct effect (i.e., the unexplained effect unrelated to variations in the mediators). The proportion of the total effect that is mediated (ratio of indirect/total effect) was also computed.

Furthermore, to evaluate whether the association of socioeconomic positions with hypertension is modified by age (<50 vs. ≥50 years) and gender (male and female), interaction terms were incorporated in the multivariable models and its significance was assessed with Wald tests. All statistical analyses were performed using Stata/IC 14 (Stata Corp., College Station, Texas, USA).

Ethical approvals from the Regional Ethical Committee, Central Norway, and Institutional Review Committee of Kathmandu University School of Medical Sciences, Nepal, were obtained. Informed consent was obtained before the start of data collection. Enumerators were trained in ethical consideration of human subject research to minimize the breach of confidentiality. The data were deidentified for analysis. The identifiers were stored for five years in a locked cabinet.

## 3. Results

The mean age of study participants was 45 years, and 51.5% were males. The majority of the participants were in their middle age (35–49 years).  Table 1 represents the distribution of sociodemographic and lifestyle factors by hypertension status. Compared to normotensives, hypertensives were generally elderly, male, employed, a member of other ethnic groups (not Dalit and Brahmin/Chettri), highly educated, less physically active, wealthy, tobacco smokers and alcohol drinkers, and consumed fewer fruits and vegetables. The distribution of socioeconomic position in relation to age, sex, and lifestyle factors are presented in Supplementary Table 1.

**Figure 2:**
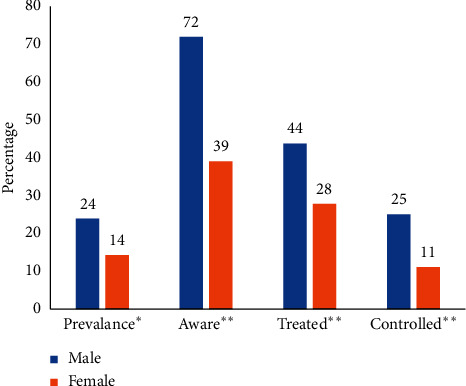
Hypertension prevalence, awareness, treatment, and control by gender. ^*∗*^*N* = total study population (260, 134M and 126F); ^*∗∗*^*N* = hypertensive (50, 32M and 18F).

Awareness, treatment, and control of hypertension: of 260 participants, 50 (23.9% males and 14.3% females) were hypertensive who either had raised blood pressure or were antihypertensive users. Sixty percent of hypertensives were aware of their status. Males were more aware and concerned about their hypertension status, were on treatment, and able to control hypertension (SBP < 140 mm Hg and DBP < 90 mm Hg) compared to females as shown in Figure 2.

Table 2 represents the SES and hypertension relationship. Compared to the low-income group, individuals belonging to middle- and high-income groups had 1.04 (95% CI, 0.54–2.01) and 1.33 (95% CI, 0.68–2.58) times more hypertension prevalence, after adjusting for age, gender, marital status, and ethnicity. Likewise, individuals who attained “less than high school” and “high school and above” had 2.02 (95% CI, 1.00–4.08) and 2.35 (95% CI, 0.88–6.29) times more prevalence of hypertension, when compared to those without formal education in an adjusted model. However, uncertainty of these point estimates is high; therefore, caution must be taken while making statistical inference. Also, the age-standardized prevalence of hypertension was found to be two times greater among individuals among higher levels of education, income, and those employed compared to their peer groups.

We found no evidence of interaction by gender and age (<50 vs ≥50 years). Furthermore, none of the lifestyle-related factors mediated the association between SES and hypertension (Supplementary Table 2 for informal assessment of mediation and Tables 3a and 3b for estimates obtained from Stata's medeff function).

## 4. Discussion

In this cross-sectional study of 260 individuals including 50 (19.2%) hypertensives, we observed a positive association between SES (education, income, and employment status) and hypertension in rural Nepal. The prevalence of hypertension was 1.33, 2.35, and 2.26 times more among individuals with higher income, higher level of education, and those employed, respectively. Sixty percent of the hypertensives were aware of their hypertension status. Moreover, there was no interaction by gender and age, and the association between SES and hypertension was not mediated via lifestyle factors.

In parallel with our findings, a 2016 health survey from Nepal reported the prevalence of hypertension to be nearly 18.9% and showed that hypertension predominated among those with a high level of education and income [19, 43]. In contrast, the 2019 STEPs survey conducted in Nepal reported a lower prevalence of hypertension among those who attained “more than secondary education” compared to those with “no or less than primary education” and no significant difference by income groups [3]. A meta-analysis study in South Asia [44] and studies in Bangladesh [45] and India [46, 47] have reported a positive association of hypertension with income and education level. Moreover, the same meta-analysis study suggested farming to be inversely associated with hypertension [44]. Similarly, a study in Vietnam reported a lower prevalence of hypertension among farmers compared to traders, construction workers, and government employees [14]. In high-income countries, an inverse association of education [48], income [11, 48, 49], and being employed [48, 50] with hypertension was reported. Studies from China [51] and Brazil [52] also reported an inverse association between education and hypertension. Previous studies from high-income countries [18] and LMICs [23, 53] including Nepal [43, 50] have reported gender differences in the association between SES and hypertension. However, we found no interaction by gender in this study.

One of the discrepancies of our study findings in relation to previous studies could be due to inconsistencies in how SES and hypertension variables were defined and the differences in the study population [53]. Furthermore, divergence in our findings with the 2019 STEPs survey could be attributable due to the large sample size and population-based sample with a higher proportion from urban setting [3]. Moreover, unlike our study findings, where we asked self-reported income to the participants, the STEPs study used a more robust and comprehensive approach to assess the income level, i.e., the household wealth index derived by the principal component analysis of household ownership of goods and facilities [3].

Nepal's epidemiological transition and adaptation of unhealthy behavior is linked with urbanization, and an early stage economic development increases the risk of developing hypertension especially among high SES [15, 25, 53]. Previous studies suggest that high SES groups dwelling in the rural setting of LMIC's such as Nepal consume high fat-containing processed food [55] and lead a sedentary lifestyle [4, 56, 57]. Thirty-three percent of Nepal's rural population, almost comparable to our study population, were multidimensionally poor [58] and not prosperous enough to reverse the SES and hypertension gradient. Nutritious diet such as fruits and vegetables is often unaffordable to poor in many countries [59], 96.7% of adults in Nepal reported insufficient intake of fruits and vegetables (<5 servings/day) [3]. Similarly, the mean dietary intake of salt, a known risk factor for hypertension, was 9.1 g/day (WHO recommends <5 g/day) [3].

Increased awareness, better accessibility to medical treatment, use of antihypertensives [60–62], and adherence to medications might lead to a lower prevalence of hypertension in high-income populations. However, for an effective management of hypertension in low-income countries such as Nepal, the significant gaps in medical treatment need to be filled [63–65]. Nevertheless, in the last few years, Nepali individuals with high income and better education have become much more aware about hypertension [3]. Interestingly, we found that Nepali individuals with high blood pressure in our study were more aware of their status; 60% knew their problems, compared to 22.2% in a national survey [3] and 43.6% in a semiurban population [66, 67].

Several studies suggest a causal pathway linking SES with hypertension through lifestyle factors [10, 29]. For instance, a study in Nepal showed that the effect of SES (education and income) on hypertension was mediated by BMI [43]. However, we did not find any mediation. Our finding that lifestyle factors did not mediate the effects of SES on hypertension could be due to weak associations observed between the mediators and the outcome and the exposures as presented in Supplementary Tables 3a and 3b. Another possibility for no mediation could be nondifferential misclassification due to the binary nature and imprecise measurement of some mediators [68, 69]. Furthermore, we cannot rule out the problem of unmeasured confounding resulting in biased estimates of SES and hypertension association towards the null [70].

Our study has two important strengths. To the best of our knowledge, this is the first study to assess whether the effect of SES on hypertension was mediated via lifestyle factors among the rural population in Nepal. Second, a validated questionnaire was used for data collection. Our study is also not without limitations. First, due to the health facility-based study with small sample size, we cannot confidently make statistical inference of our findings to the population, i.e., the uncertainty of point estimates was considerably higher. Second, due to the cross-sectional nature of the study design, we cannot rule out the possibility of reverse causality. Third, imprecise construction of some of the variables in the mediation analysis might have resulted in overestimating the direct effect sizes and underestimating the indirect effect sizes [68, 69]. Fourth, due to self-reported data, our results might be tied to recall bias [71]. Fifth, we did not have sufficient power to explore the role of important mediator dietary salt intake because majority consumed little or right amount of salt. Furthermore, we cannot rule out the possibility of residual confounding due to coarse adjustment of confounders such as ethnicity.

In summary, we found that awareness of hypertension status was high in Nepal's rural setting, and socioeconomic determinants were positively associated with hypertension, with no evidence of mediation by lifestyle factors. We believe large longitudinal studies are required to replicate our findings in the rural setting of Nepal. Studies are also warranted to assess the availability, adherence, and affordability of hypertension particularly in a rural setting of Nepal. This would help in preparing a roadmap for the hypertensive prevention program in rural Nepal.

## Figures and Tables

**Table 1:** Distribution of sociodemographic, lifestyle, and SES factors by hypertension status.

	Total *N* = 260 (*N*)	Normotensive *N* = 210 (*N*%)	Hypertensive *N* = 50 (*N*%)
Gender			
Male	134	102 (76.1)	32 (23.9)
Female	126	108 (85.7)	18 (14.3)
Age groups (categories)			
18–34 years	77	67 (87.0)	10 (13.0)
35–49 years	88	70 (79.5)	18 (20.5)
50–65 years	55	45 (81.8)	10 (18.2)
66 years and above	40	28 (70.0)	12 (30.0)
Age in years, mean (±SD)	45 (±16.4)	44.2 (±16.1)	50.8 (±16.7)
Marital status			
Unmarried	38	31 (81.6)	7 (18.4)
Married	222	179 (80.6)	43 (19.4)
Ethnicity			
Brahmin/Chettri	173	141 (81.5)	32 (18.5)
Dalits	35	26 (74.3)	9 (25.7)
Others	52	43 (82.7)	9 (17.3)
Education			
No formal education	113	94 (83.2)	19 (16.8)
Less than high school	106	85 (80.2)	21 (19.8)
High school or more	41	31 (75.6)	10 (24.4)
Income			
Low income	87	72 (82.8)	15 (17.2)
Middle income	87	72 (82.8)	15 (17.2)
High income	86	66 (76.7)	20 (23.3)
Annual income median (IQR), NRS	16,733 (35,994)	16333 (31,833)	26286 (46,154)
Employment status			
Unemployed	59	47 (79.7)	12 (20.3)
Farming	128	111 (86.7)	17 (13.3)
Employed	73	52 (71.2)	21 (28.8)
Lifestyle factors			
Tobacco use			
Never	108	88 (81.5)	20 (18.5)
Current	60	50 (83.3)	10 (16.7)
Former	92	72 (78.3)	20 (21.7)
Alcohol intake			
Never	195	158 (81.0)	37 (19.0)
Low (<1 glass per week)	12	10 (83.3)	2 (16.8)
Moderate (1–3 glass per week)	14	9 (64.3)	5 (35.7)
High (>3 glass per week)	39	33 (84.6)	6 (15.4)
Physical activity			
MET^*∗*^ < 600 min/week	26	18 (69.2)	8 (30.8)
MET ≥ 600 min/week	234	192 (82.1)	42 (17.9)
Fruits and vegetables servings			
<2 servings per day	35	27 (77.1)	8 (22.9)
2–4 servings per day	204	164 (80.4)	40 (19.6)
>4 servings per day	21	19 (90.5)	2 (9.5)
Body mass index^*∗∗*^, kg/m^2^			
Under weight (<18.5)	36	32 (88.9)	4 (11.1)
Normal weight (18.5–24.9)	160	138 (86.2)	22 (13.8)
Overweight (25.0–25.9)	52	33 (63.5)	19 (36.5)
Obesity (≥30)	12	7 (58.3)	5 (41.7)
BMI, mean (SD)	22.5 (3.9)	21.9 (3.54)	24.9 (4.37)

^*∗*^MET is the ratio of the rate of energy expended during an activity to the rate of energy expended at rest. ^*∗∗*^Defined based on the WHO criteria.

**Table 2:** Relationship between socioeconomic status and hypertension.

Socioeconomic factors	Hypertension, *N* (%)	Age standarised^a^ hypertension prevalence % (95% CI)	Model 1^b^		Model 2^d^	
			PR^c^ (95% CI)	*P* value	PR^c^ (95% CI)	*P* value
Income						
Low	15 (17.2%)	10 (4%–15%)	Ref		Ref	
Middle	15 (17.2%)	14 (7%–21%)	0.91 (0.47–1.76)	0.778	1.04 (0.54–2.01)	0.908
High	20 (23.3%)	26 (17%–36%)	1.06 (0.54–2.11)	0.845	1.33 (0.68–2.58)	0.407
Education						
No formal education	19 (16.8%)	10 (4%–15%)	Ref		Ref	
Less than high school	21 (19.8%)	16 (9%–23%)	0.98 (0.54–1.77)	0.951	2.02 (1.00–4.08)	0.049
High school and above	10 (24.4%)	24 (11%–38%)	0.91 (0.40–2.03)	0.812	2.35 (0.88–6.29)	0.089
Employment status						
Unemployed	12 (20.3%)	17 (7%–26%)	Ref (1.0)		Ref (1.0)	
Farming	17 (13.3%)	10 (3%–17%)	0.66 (0.33–1.32)	0.239	1.00 (0.48–2.07)	0.999
Employed	21 (28.8%)	21 (11%–32%)	1.44 (0.73–2.82)	0.293	2.26 (1.02–5.05)	0.046

^a^Standardised to the World Health Organization standard population; ^c^PR, prevalence ratio. ^b^Model 1, unadjusted. ^d^Model 2, adjusted for age (continuous), gender (male/female), marital status (married/unmarried), and ethnicity (Brahmin/Chettri/Dalits/others).

## Data Availability

The data used to support the findings of this study are available from the corresponding author upon request and shared with approval from Regional Ethical Committee, Central Norway.

## Supplementary Materials

Supplementary Materials Supplementary Table 1 presents the distribution of the socioeconomic position in relation to age, sex, and lifestyle factors. Supplementary Table 2 presents the informal assessment of mediating role of life style factors in SES and hypertension relationship using logistic regression. Supplementary Table 3a presents the results-mediated effects of income on hypertension via lifestyle factors using medeff command in Stata. Supplementary Table 3b presents the results mediated effects of education on hypertension via lifestyle factors using medeff command in Stata. Show cards are shown to the respondents during data collection; the show cards used were same as the one used in the Noncommunicable Diseases Risk Factors: STEPS Survey Nepal 2013 to identify the type of tobacco the respondents used, to determine the amount of alcohol the respondents consumed, to identify the type of fruits the respondents ate, and to identify the type of physical activity the respondents were engaged in.
