# Supplementary material for: Socioeconomic Status and Its Relation to Hypertension in Rural Nepal
Source: Int J Hypertens. 2021 Aug 28;2021:5542438. doi: 10.1155/2021/5542438 (PMC8419497; doi:10.1155/2021/5542438)
Supplement: Supplementary Materials — Supplementary Table 1 presents the distribution of the socioeconomic position in relation to age, sex, and lifestyle factors. Supplementary Table 2 presents the informal assessment of mediating role of life style factors in SES and hypertension relationship using logistic regression. Supplementary Table 3a presents the results-mediated effects of income on hypertension via lifestyle factors using medeff command in Stata. Supplementary Table 3b presents the results mediated effects of education on hypertension via lifestyle factors using medeff command in Stata. Show cards are shown to the respondents during data collection; the show cards used were same as the one used in the Noncommunicable Diseases Risk Factors: STEPS Survey Nepal 2013 to identify the type of tobacco the respondents used, to determine the amount of alcohol the respondents consumed, to identify the type of fruits the respondents ate, and to identify the type of physical activity the respondents were engaged in. [file 5542438.f1.pdf]

Supplementary Tables

Supplementary Table 1: Distribution of socioeconomic position in relation to age, sex and lifestyle factors

	**Education**			**Income**		
	No formal education (N=113)	Less than high school (n=106)	High School or more (n=41)	Low income (n=87)	Middle income (n=87)	High income (n=86)
**Sex**						
Male	36 (31.9)	71 (67.0)	27 (65.8)	51 (58.6)	41 (47.1)	34 (39.5)
Female	77 (68.1)	35 (33.0)	14 (34.2)	36 (41.4)	46 (52.9)	52 (60.5)
**Age groups in years**						
18-34	12 (10.6)	41 (38.7)	24 (58.5)	15 (17.3)	23 (26.4)	39 (45.3)
35-49	33 (29.2)	41 (38.7)	14 (34.2)	24 (27.6)	36 (41.9)	28 (32.7)
50-65	35 (31.0)	17 (16.0)	3 (7.3)	21 (24.1)	19 (21.8)	15 (17.4)
66 and above	33 (29.2)	7 (6.6)	0 (0.0)	27 (31.0)	9 (10.3)	4 (4.6)
Age in years, Mean (SD)	54.8 (15.7)	40.1 (13.5)	33.2 (10.1)	52.8 (17.3)	44.8 (14.7)	38.6 (14.1)
**Tobacco use**						
Never	40 (35.4)	40 (37.8)	28 (68.3)	33 (37.9)	31 (35.6)	44 (51.2)
Current	21 (18.6)	33 (31.1)	6 (14.6)	19 (21.8)	24 (27.6)	17 (19.8)
Former	52 (46.0)	33 (31.1)	7 (17.1)	35 (40.2)	32 (36.8)	25 (29.1)
**Alcohol intake**						
Never	95 (84.1)	71 (66.9)	29 (70.7)	71 (81.6)	63 (72.4)	61 (70.9)
Low (<1 glass per week)	2 (1.8)	6 (5.7)	4 (9.8)	4 (4.6)	4 (4.6)	4 (4.7)
Moderate (1-3 glass per week)	7 (6.2)	6 (5.7)	1 (2.4)	4 (4.6)	7 (8.1)	3 (3.5)
High (>3 glass per week)	9 (7.9)	23 (21.7)	7 (17.1)	8 (9.2)	13 (14.9)	18 (20.9)
**Physical activity**						

MET* < 600 min/week	14 (12.4)	10 (9.4)	2 (4.9)	12 (13.8)	4 (4.6)	10 (11.6)
MET ≥ 600 min/week	99 (87.6)	96 (90.6)	39 (95.1)	75 (86.1)	83 (95.4)	76 (88.4)
**Fruits and vegetables servings**						
<2 servings per day	10 (8.8)	17 (16.0)	8 (19.5)	9(10.3)	11 (12.7)	15 (17.5)
2-4 servings per day	95 (84.1)	80 (75.5)	29 (70.7)	71 (81.6)	67 (77.0)	66 (76.7)
>4 servings per day	8 (7.1)	9 (8.5)	4 (9.8)	7 (8.1)	9 (10.3)	5 (5.8)
**Body Mass Index**** kg/m^2,						
Normal weight	66 (58.4)	72 (67.9)	22 (53.7)	52 (59.8)	59 (67.8)	49 (57.0)
Under weight	26 (23.0)	8 (7.5)	2 (4.9)	21 (24.1)	9 (10.3)	6 (7.0)
Overweight	17 (15.0)	20 (18.9)	15 (36.6)	10 (11.5)	15 (17.3)	27 (31.4)
Obesity	4 (3.5)	6 (5.7)	2 (4.9)	4 (4.6)	4 (4.6)	4 (4.6)
BMI kg/m^2, mean (SD)	21.5 (3.8)	23.1 (3.8)	24.0 (3.7)	21.2 (3.8)	22.5 (3.7)	23.8 (3.8)

** MET is the ratio of the rate of energy expended during an activity to the rate of energy expended at rest. **defined based on WHO criteria*

Supplementary Table 2: Informal assessment of mediating role of life style factors using logistic regression

**Socioeconomic Variables**	**Model 1[a] OR[b] (95%CI)**	**Model 1 & Tobacco use OR[b] (95%CI)**	**Model 1 & Alcohol intake OR[b] (95%CI)**	**Model 1 & Physical activity OR[b] (95%CI)**	**Model 1 & Fruits and vegetables servings OR[b] (95%CI)**	**Model 1 & BMI OR[b] (95%CI)**	**Model 2[c] OR[b] (95%CI)**
**Income**							
Low	Ref (1.0)	Ref (1.0)	Ref (1.0)	Ref (1.0)	Ref (1.0)	Ref (1.0)	Ref (1.0)
Middle	1.1 (0.4 - 2.6)	1.0 (0.4-2.6)	1.1(0.5-2.6)	1.0 (0.4 - 2.4)	1.1 (0.4-2.6)	0.9 (0.4-2.3)	0.9 (0.3 -2.2)
High	1.5 (0.6-3.7)	1.4 (0.9-6.4)	1.5 (0.6-4.0)	1.2 (0.5-3.2)	1.4 (0.6-3.7)	1.1 (0.4-2.9)	1.0 (0.4-2.7)
**Education**							
No formal education	Ref (1.0)	Ref (1.0)	Ref (1.0)	Ref (1.0)	Ref (1.0)	Ref (1.0)	Ref (1.0)
Less than high school	2.5 (0.9-6.6)	2.4 (0.9-6.4)	2.5 (1.0-6.7)	2.7 (1.0-7.5)	2.5 (0.9-6.5)	2.0 (0.7-5.5)	2.3 (0.8-6.5)
High school and above	3.2 (0.8-12.5)	3.1 (0.7-12.0)	3.3 (0.9-13.0)	3.4 (0.9-13.2)	3.3 (0.9-13.0)	2.4 (0.6-9.7)	2.7 (0.7-10.9)
**Employment status**							
Unemployed	Ref (1.0)	Ref (1.0)	Ref (1.0)	Ref (1.0)	Ref (1.0)	Ref (1.0)	Ref (1.0)
Farming	1.0 (0.4-2.7)	1.0 (0.4-2.6)	1.0 (0.4-2.7)	1.4 (0.5-2.7)	1.0 (0.4-2.7)	1.3 (0.5-3.6)	1.8 (0.6-5.4)
Employed	2.9 (0.9-9.1)	2.8 (0.9-8.77)	2.9 (0.9-9.4)	3.2 (1.0-10.6)	2.8 (0.9-8.9)	2.9 (0.9-9.5)	3.3 (1.0-11.4)

[a] Adjusted for age, sex, marital status and ethnicity [b]OR = Odds ratio; *significant <0.05

[c]Adjusted for age, gender, marital status, ethnicity, physical activity, tobacco use, alcohol intake, vegetable and fruits servings and BMI

Supplementary Table 3a: Mediated effects of income on hypertension via lifestyle factors

Mediators	Effect of income on mediators Beta (95% CI)	Effect of mediators on hypertension Beta (95% CI)	Effect of income on hypertension Beta (95% CI)	Indirect effect Mean (95% CI)	Direct effect Mean (95% CI)	Total Effect Mean (95% CI)	Prop. of Total Effect mediated Mean (95% CI)
Tobacco use	-8.49e-06 (-0.000 - 3.41e-06)	-0.240 (-1.090 - 0.610)	5.32e-06 (-3.38e-06 - 0.000)	-0.000 (-0.003 - 0.002)	6.57e-07 (-4.94e-07 - 1.62e-06)	-0.000 (-0.003 - 0.002)	0.242 (-18.440 - 21.501)
Alcohol intake	1.12e-06 (-4.99e-06 - 7.23e-06)	-0.011 (-0.033 - 0.012)	6.16e-06 (-2.84e-06 - 0.000)	-0.000 (-0.000 - 0.000)	7.73e-07 (-4.29e-07 - 1.75e-06)	-0.000 (-0.000 - 0.000)	0.252 (-27.069 - 19.687)
Physical activity	-9.02e-07 (-0.000 - 9.09e-06)	-0.000 (-0.000 - -2.56e-06)	4.87e-06 (-3.65e-06 - 0.000)	-1.98e-07 (-5.64e-07 - 3.34e-08)	7.35e-07 (-6.47e-07 - 1.87e-06)	5.37e-07 (-8.97e-07 – 1.68e-06)	-0.224 (-4.598 - 3.525)
Fruits and vegetables servings	5.38e-06 (-1.22e-06 - 0.000)	0.204 (-0.542 - 0.949)	5.50e-06 (-3.29e-06 - 0.000)	-0.001 (-0.002 - 0.005)	6.76e-07 (-4.94e-07 - 1.63e06)	0.000 (-0.002 - 0.005)	-0.398 (-22.349 - 8.736)
BMI	6.48e-06 (-2.01e-06-0.000)	1.375 (-0.639-2.111)	4.30e-06 (-4.37e-06-0.000)	0.007 (0.000 - 0.016)	4.93e-07 (-6.10e-07 - 1.41e-06)	0.007 (0.000 - 0.016)	1.053 (0.389 - 6.422)

Supplementary Table 3b: Mediated effects of education on hypertension via lifestyle factors

Mediators	Effect of education on mediators beta (95% CI)	Effect of mediators on hypertension beta (95% CI)	Effect of education on hypertension beta (95% CI)	Indirect effect Mean (95% CI)	Direct effect Mean (95% CI)	Total Effect Mean (95% CI)	Prop. of Total Effect mediated Mean (95% CI)
Tobacco use	-0.555 (-1.217 - 0.106)	-0.240 (-1.090 - 0.610)	-0.623 (-0.047 - 1.290)	0.002 (-0.008 - 0.012)	0.079 (-0.007 - 0.156)	0.081 (-0.004 - 0.157)	0.020 (-0.062 - 0.135)
Alcohol intake	-0.278 (-0.890 - 0.334)	-0.011 (-0.033 - 0.012)	-0.649 (-0.018 - 1.316)	0.000 (-0.000 - 0.000)	0.085 (-0.003 - 0.161)	0.085 (-0.003 - 0.161)	0.001 (-0.003 - 0.004)
Physical activity	-1.009 (-2.280 - 0.262)	-0.000 (-0.000 - -2.56e-06)	0.572 (-0.097 - 1.241)	2.99e-07 (-3.27e-07 - 1.26e-06)	0.088 (-0.017 - 0.175)	0.088 (-0.017 - 0.175)	3.13e-06 (-0.000 - 0.000)
Fruits and vegetables servings	-0.267 (-0.794 - 0.259)	0.204 (-0.542 - 0.949)	0.652(-0.017 - 1.321)	-0.001 (-0.010 - 0.005)	0.083 (-0.002 - 0.160)	0.082 (-0.002 - 0.158)	-0.012 (-0.075 - 0.040)
BMI	0.943 (0.356 - 1.530)	1.365 (0.639 - 2.111)	0.416 (-0.270 - 1.103)	0.033 (0.010 - 0.061)	0.050 (-0.037 - 0.126)	0.083 (-0.005 - 0.159)	0.373 (-1.085 - 2.108)

Show cards used during Data collection

Source: Non-Communicable diseases Risk Factors: STEPS Survey Nepal 2013

1. Tobacco Products

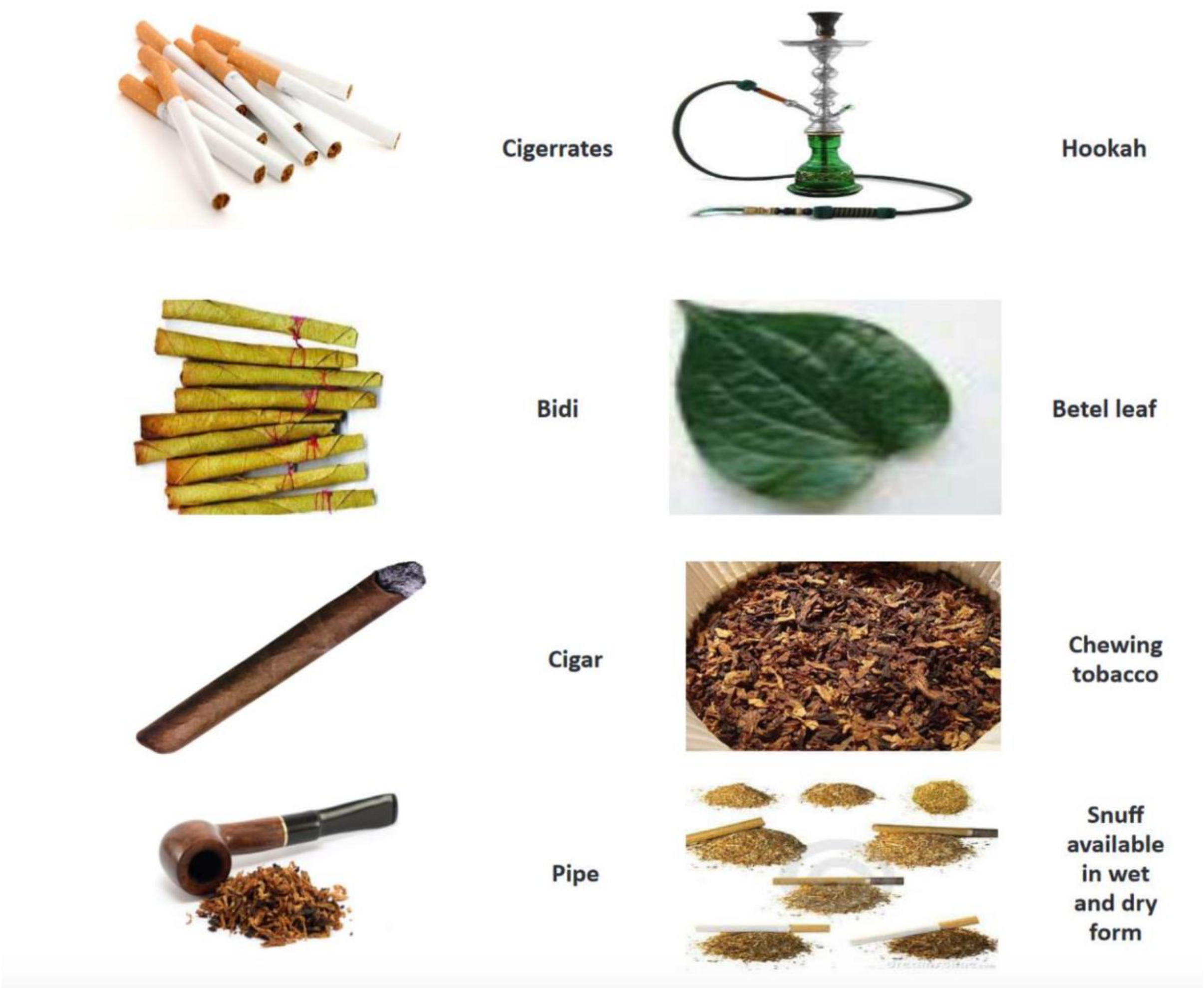

2. Alcohol Vessel

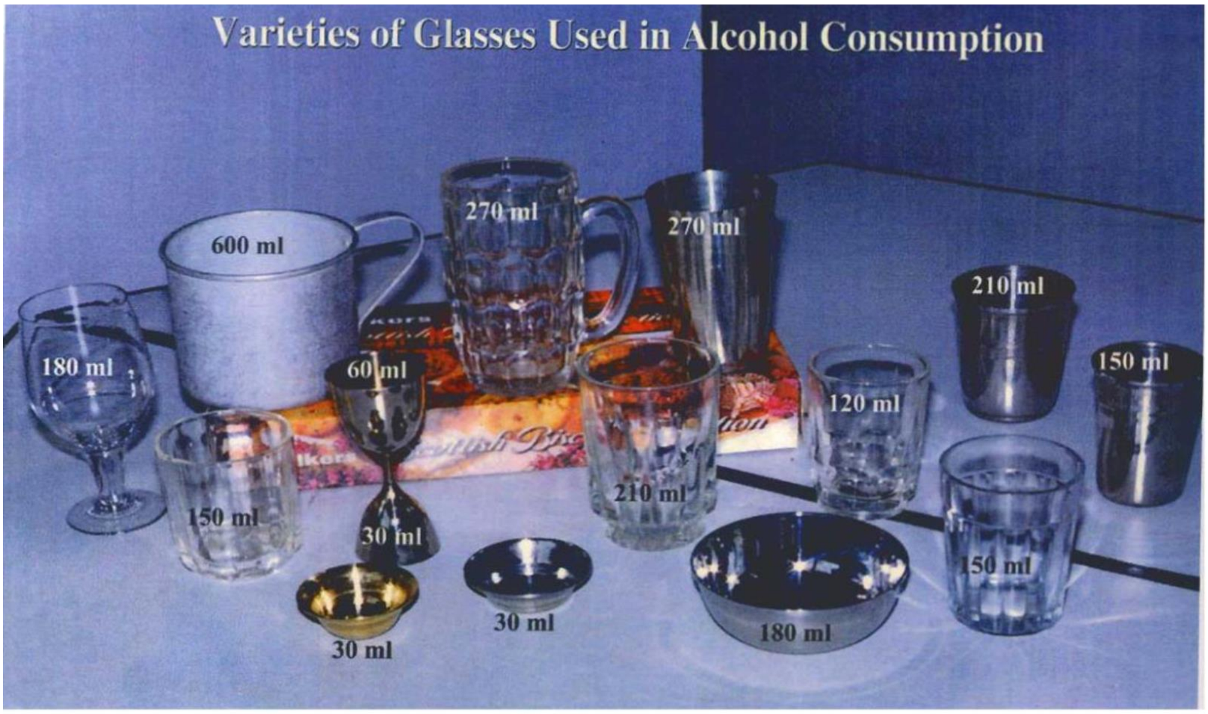

3. Diet

**JACK FRUIT** 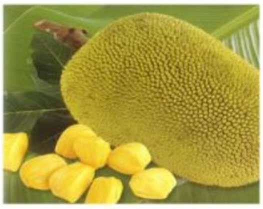	**BANANA** 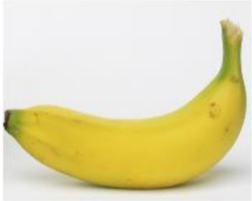	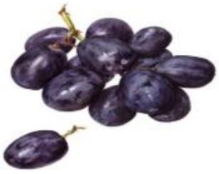 **GRAPES**	**MANGO** 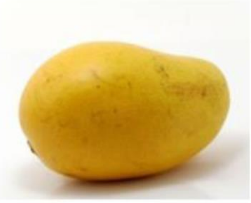
**APPLE** 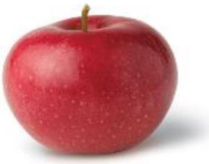	**ORANGE** 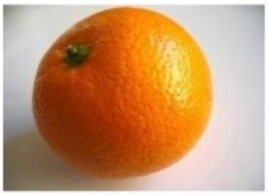	**PEACH** 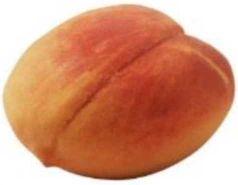	**PEAR** 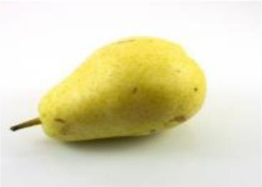
**STRAWBERRIES** 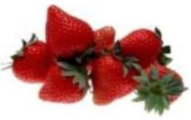	**WATERMELON** 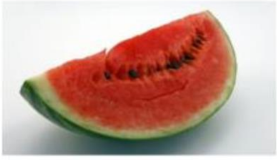	**PINEAPPLE** 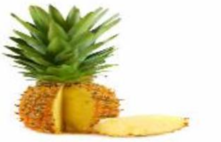	**LYCHEES** 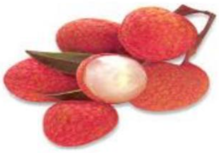
**POMELO** 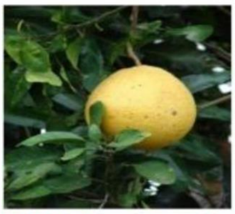	**PLUM** 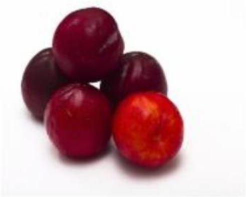	**GRAPEFRUIT** 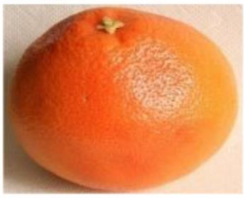	**GUAVA** 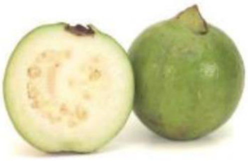

Serving size: One standard serving = 80 grams

4. Typical Physical activities

Vigorous activities

Moderate activities

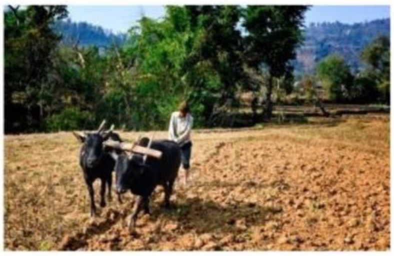

Ploughing field

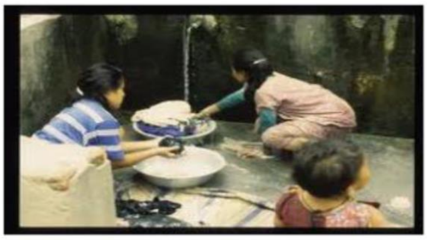

Housework and domestic chores

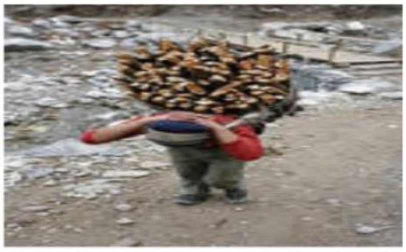

Carrying heavy load

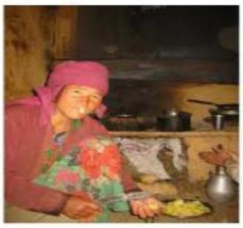

Kitchen Work

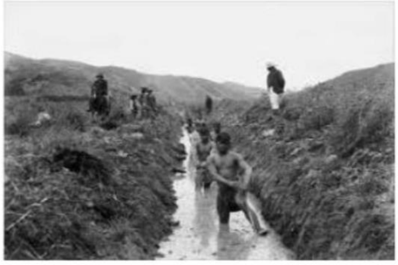

Digging ditch

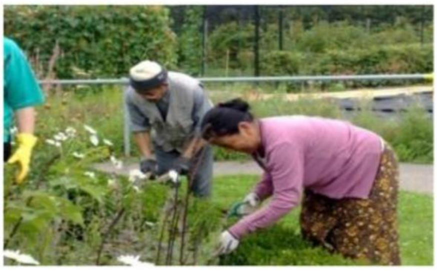

Gardening

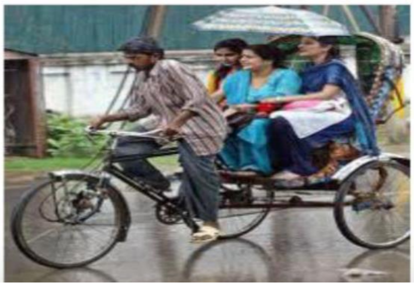

Cycle rickshaw driving

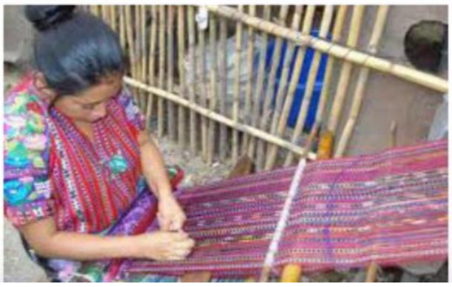

Weaving
